# New Editing Tools for Gene Therapy in Inherited Retinal Dystrophies

**DOI:** 10.1089/crispr.2021.0141

**Published:** 2022-06-08

**Authors:** Juliette Pulman, José-Alain Sahel, Deniz Dalkara

**Affiliations:** ^1^ Sorbonne Université, INSERM, CNRS, Institut de la Vision, Paris, France.; ^2^ Department of Ophthalmology, The University of Pittsburgh School of Medicine, Pittsburgh, Pennsylvania, USA.; ^3^ CHNO des Quinze-Vingts, INSERM-DGOS CIC 1423, Paris, France.; ^4^ Fondation Ophtalmologique Rothschild, Paris, France.

## Abstract

Inherited retinal dystrophies (IRDs) are a heterogeneous group of diseases that affect more than 2 million people worldwide. Gene therapy (GT) has emerged as an exciting treatment modality with the potential to provide long-term benefit to patients. Today, gene addition is the most straightforward GT for autosomal recessive IRDs. However, there are three scenarios where this approach falls short. First, in autosomal dominant diseases caused by gain-of-function or dominant-negative mutations, the toxic mutated protein needs to be silenced. Second, a number of IRD genes exceed the limited carrying capacity of adeno-associated virus vectors. Third, there are still about 30% of patients with unknown mutations. In the first two contexts, precise editing tools, such as CRISPR-Cas9, base editors, or prime editors, are emerging as potential GT solutions for the treatment of IRDs. Here, we review gene editing tools based on CRISPR-Cas9 technology that have been used *in vivo* and the recent first-in-human application of CRISPR-Cas9 in an IRD.

## Introduction

Inherited retinal dystrophies (IRDs) are a heterogeneous group of diseases that affect more than 2 million people worldwide.^1^ Clinical presentations are very heterogeneous, with variable symptoms (either isolated or syndromic), age of onset, and, in most cases, severity. IRDs are genetically very heterogeneous, with almost 300 genes reported (https://sph.uth.edu/retnet/) and often with multiple mutations for each gene. They can be transmitted by all modes of inheritance: autosomal recessive (ar), autosomal dominant (ad), X-linked, or mitochondrial.

Gene therapy (GT) has emerged as an exciting treatment modality with the potential to provide long-term benefit to patients. Today, gene addition (also referred to as gene supplementation or replacement) is the most straightforward GT for arIRDs and is rapidly gaining ground in the clinic. Gene addition therapy for *RPE65* deficiency, marketed under the name Luxturna, is the first successful implementation of adeno-associated virus (AAV)-mediated GT in ophthalmology. Luxturna is destined for the treatment of retinitis pigmentosa (RP) or Leber congenital amaurosis (LCA) associated with *Rpe65* mutations.^2,3^

Despite the successful application of gene addition in this type of rare monogenic recessive IRD, there are three scenarios where this approach falls short. First, in ad diseases that are caused by gain-of-function or dominant-negative mutations leading to toxicity, gene addition cannot be used. Second, a number of IRD genes exceed the limited carrying capacity of AAV vectors of <5 kB. Third, there are still about 30% of patients with unknown mutations that cannot be treated with this type of approach.

In this context, precise editing tools, such as CRISPR-Cas9, are emerging as a potential GT solution for the two first scenarios, opening new possibilities for treatments of IRDs. Due to its immunoprivileged environment and relative isolation from other organs, the eye is at the forefront of innovative therapeutics and is a good indicator to know where the future major advances will be in GT. In this review, we explore gene editing tools based on CRISPR-Cas9 technology that have been used *in vivo* in the eye, and we highlight those that are already in clinical application. We also focus on the potential future uses and limits of CRISPR-Cas9 and other new editing tools in IRDs.

## Gene Editing Tools Used for the Treatment of IRDs

Gene editing tools have aroused interest for years for their potential utility in GT. Zinc finger nucleases and transcription activator-like effector nucleases (TALENs) were the first-generation nucleases to be used in gene editing.^4–6^ They achieve sequence-specific DNA-binding via protein–DNA interactions. TALEN was, for example, used to correct *Crb1* in mice by homology-directed repair (HDR).^7^ However, their complex design makes them more difficult to engineer new versions, reducing their potential applicability toward use in research and therapy.^4,8^

CRISPR-Cas9 is a powerful tool that can precisely and easily edit a specific sequence of DNA. The meteoric rise in its use since its application in mammalian cells in 2013^9^ is due to the ease of design and handling, now make it a promising tool for GT. Here, we review how CRISPR-Cas9 has been used *in vivo*, mainly in murine models but also in its first use in humans to treat IRDs as part of an ongoing clinical trial (NCT03872479). We also discuss the use of new editing tools, namely base editors (BEs) and prime editors (PEs), and the critical steps preceding their clinical application in IRDs.

### CRISPR-Cas9 for the treatment of IRDs

Recently, the idea of using targeted genome editors has been set forth as an alternative means to achieve therapeutic benefit in IRDs. In particular, the CRISPR system brought genome editing into the mainstream with an increasing number of applications.^10^

CRISPR-Cas9, derived from a bacterial adaptive immune system, is a simple, easy-to-use, and highly specific gene editing tool. In the CRISPR-Cas9 system, a Cas9 endonuclease is directed to a specific DNA region via a guide RNA (gRNA). The Cas9 endonuclease then induces a site-specific double-strand break (DSB). The site-specific alteration of DNA activates multiple DNA repair mechanisms: non-homologous end joining (NHEJ) rapidly ligates DNA ends without processing, which can lead to small insertions or deletions (indels) and gene disruption, whereas HDR uses a DNA template. HDR can be used to induce a specific modification at a specific site.^11^

Because the gRNA targets a specific site, CRISPR-Cas9 genome editing can be easily directed to virtually any genomic site by delivering the complementary gRNA sequence along with the Cas9 endonuclease.^12^ As the design of the gRNA is simple, it is relatively easy to target a new gene or a new mutation using the same tool.

**FIG. 1.**
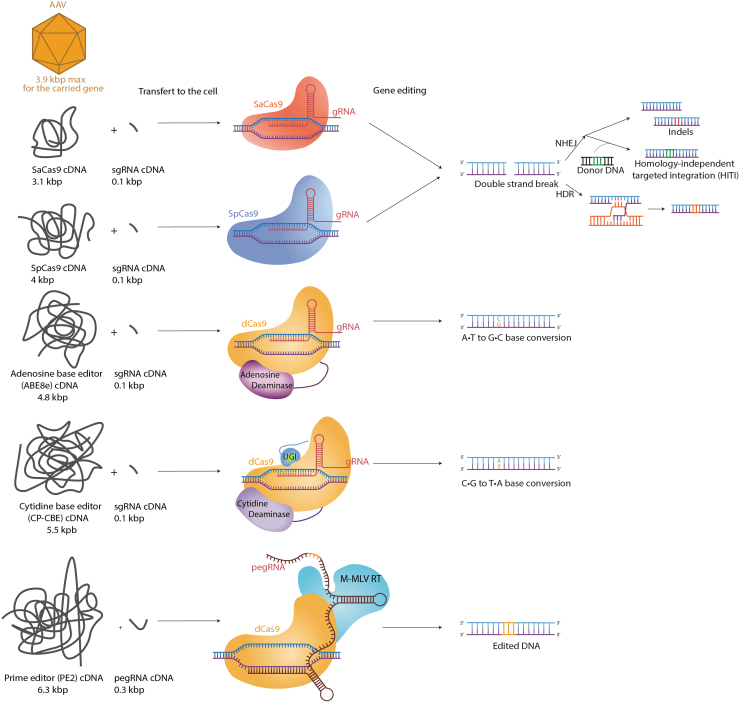
Characteristics and mechanism of several CRISPR-Cas9 gene editing tools. Adeno-associated virus capacity is limited to maximum of 3.9 kb for the carried gene. However, the sizes of the cDNA and proteins are increasing with the improvements and changes in the different gene editing tools. ABE8E cDNA size from Ritcher *et al*.^98^ CP-CBEE cDNA size from Huang *et al*.^99^ PE cDNA size from Anzalone *et al*.^46^

**FIG. 2.**
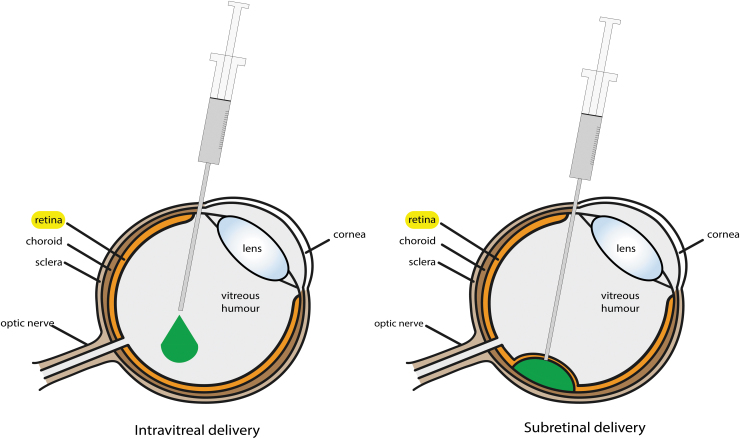
Schematic figure of the eye, demonstrating the possible routes of delivery to introduce a gene editing system into the eye to target the retina (highlight in *yellow*) for gene therapy to treat IRDs.

Multiple Cas proteins have been discovered and studied, with different sizes, efficacy, and recognition motifs. In control mice, *Streptococcus pyogenes* Cas9 (SpCas9) was the most efficient one using an AAV2-7m8 vector in retinal cells *in vivo*.^13^ However, it is difficult to fit SpCas9 into one AAV. And a dual AAV system can be problematic when translating to the clinic. Therefore, *Streptococcus aureus* Cas9 (SaCas9) is often used, as it can fit into a single AAV along with its gRNAs (Fig. 1). CRISPR-Cas9 can be delivered to the retina by subretinal injection or by intravitreal injection (Fig. 2).

The first to send CRISPR-Cas9 in the eye for therapeutic use, in 2016, Bakondi *et al*. injected a plasmid containing Cas9 and gRNA subretinally in combination with electroporation in a rat model of IRD with mutation Rho.S334ter.^14^ Using a gRNA targeting an allele-specific single nucleotide substitution unique to the RhoS334 allele, they obtained an allele-specific disruption of Rho.S334, which led to a 35% improvement in the visual acuity of the treated eye compared to controls, observed by optokinetic tests. However, no difference in visual function was detected by electroretinography (ERG).^14^

It has also been found that subretinal electroporation of a CRISPR-Cas9 plasmid expressing two gRNAs into Rho.P23H transgenic mice leads to specific disruption of the mutant allele and reduction of the mutant RHO protein.^15^ Later, two articles confirmed the feasibility of an allele-specific disruption of Rho.P23H, one with a plasmid DNA and another with a dual AAV9 variant delivery (AAV9-PHP.B), both showing a reduction of photoreceptor degeneration with a partial preservation of the outer nuclear layer (ONL) thickness.^16,17^ Delivery using an AAV vector leads to improved retinal function measurable by ERG.^17^

Another study used a mutation-dependent approach for the second most prevalent mutation P347S of *RHO*. They obtained around 10% of indels in murine photoreceptors after subretinal delivery of a dual AAV2/8 carrying SpCas9 and its gRNA, which leads to a significant improvement of the ERG b-wave amplitude in transgenic mice.^18^

All these approaches rely on mutation-specific gRNAs. They specifically disrupt the mutant allele using CRISPR-Cas9 to reduce its toxic effect and rely on the wild-type (wt) allele to express sufficient amounts of protein. However, in some cases, this might not be sufficient due to haploinsufficiency. Moreover, IRDs are genetically very heterogeneous, and there are often multiple different mutations, even in the same gene. For example, there are more than 100 dominant mutations in the *RHO* gene (see https://sph.uth.edu/retnet/home.htm), and it seems difficult to generate a sequence-specific inhibitor for each mutation at a large scale for a putative therapy.

Consequently, mutation-independent methods have been tested, with the disruption of both alleles, and with an additional gene supplementation. For example, Tsai *et al*. used a dual AAV2/8 vector with saCas9 and a double gRNA to disrupt both alleles of *RHO* in a murine model carrying the RHO.P23H mutation, with a gene addition of the wt *RHO* cDNA. They obtained an improvement of the ONL thickness and a functional improvement of the photoreceptors.^19^ Recently, this group used the same strategy of suppression and replacement but with a dual AAV8 vector in a new *h*RHO^C110R^/*h*RHO^wt^ humanized murine model of rod-cone degeneration. They significantly hamper photoreceptor degeneration for at least 6 months, showing that this suppression and replacement strategy could be used also for other adIRDs.^20^

In parallel, another interesting use of CRISPR-Cas9 was developed to treat RP in a gene-independent manner. *Nrl* is responsible for rod fate determination during photoreceptor development. Therefore, disrupting *Nrl* by CRISPR-Cas9 leads to the reprogramming of rods to cone-like photoreceptors.^21^ In RP, most of the mutations are found in rod-expressed genes, leading to primary rod photoreceptor death and degeneration. By changing the rod cell type to cones that do not express the mutant gene, negative effects of the mutation can be circumvented, and the cone photoreceptor cells can survive longer,^22^ restoring visual function in two murine models of RP.^23^

McCullough *et al*. show that editing with CRISPR-Cas9 in somatic photoreceptor cells is transferable to nonhuman primates. They targeted *GUCY2D* using saCas9 delivered by an AAV5 and demonstrated reduced retGC1 expression.^24^ Shortly after, Maeder *et al*. also used nonhuman primates as a model to complete the preclinical proof of concept for the use of CRISPR-Cas9 for therapy in LCA type 10 (LCA10), an IRD causing severe childhood blindness caused by *CEP290* mutations. The most common mutation is IVS26 (c.2991 + 1655A>G), a point mutation located within an intron. It creates a novel splice donor site, resulting in the inclusion of 128 bp and creating a premature stop codon.

EDIT-101 is a therapy that specifically targets this mutation using the CRISPR-SaCas9 technology and two specific gRNAs packaged into an AAV5. When injected subretinally in both mice and nonhuman primates, EDIT-101 achieved an editing efficiency of >10%, which could in theory be sufficient for a therapeutic effect, since approximately 10% functional foveal cone photoreceptors would be sufficient for a good visual acuity.^25^

EDIT-101 is now in Phase I/II clinical trials (NCT03872479), and recently positive outcomes have been reported in terms of both visual outcomes and safety (from the XIX International Symposium on Retinal Degeneration, 2021, Mark Pennesi et al., “BRILLIANCE: A Phase 1/2 Single Ascending Dose Study of EDIT-101, an *in vivo* CRISPR Gene Editing Therapy, in CEP290-Related Retinal Degeneration”). Today, CRISPR-Cas9 is the most advanced gene-editing tool, compared to BEs or PEs, to proceed to clinical applications. And based on current results promises, CRISPR will continue to bring new possibilities to treat IRDs.

Most of the currently reported studies transport the CRISPR-Cas9 system's DNA via an AAV. However, as AAV delivery of CRISPR-Cas9 leads to the permanent expression of Cas9 protein,^26^ this might have adverse effects on the rest of the genome^27^ as well as potentially eliciting immune reactions to the microbial Cas9 protein.^28^ Unlike gene replacement therapy, the CRISPR-Cas9 and its gRNA only need to be present for a limited amount of time to be functional. Therefore, transient expression of CRISPR-Cas9 using either mRNA or ribonucleoprotein (complexes of Cas9 protein with its gRNA) using a nonviral delivery system arose as a possibility to deliver this system into cells.

Lipids are currently the most developed nonviral delivery vectors.^29^ However, they have as yet only been used in an age-related macular degeneration murine model^30,31^ and not for IRDs. Also, nanoparticles composed of thin glutathione (GSH)-cleavable covalently crosslinked polymer coating and decorated with the all-trans retinoic acid were also used to deliver CRISPR-Cas9 to murine eyes *in vivo* and led to >4% gene editing in retinal pigment epithelium (RPE) cells,^32^ showing its feasibility but also the limited efficacy of nonviral vectors.

In conclusion, AAV is the main vector system today for the delivery of CRISPR-Cas9 into retinal cells. However, due to possible long-term effects of the permanent expression of CRISPR-Cas9, nonviral delivery systems of transient Cas9 are being explored.^32^ However, their efficacy is still limited, and their toxicity needs to be investigated.

### Improving CRISPR-Cas9 for the treatment of IRDs

In the previous presented analyses, Cas9 is mainly used for its DSB capacity, and the main repairing pathway exploited is NHEJ, which leads to indels and gene inactivation. HDR, another repair pathway, can specifically introduce a defined genomic change using a template DNA sequence. But HDR only functions in the late S-G2 phase and therefore in dividing cells. So, it is not a therapeutically viable mechanism to induce DNA repair in post-mitotic retinal cells and neurons in general. Nevertheless, CRISPR-CjCas9-mediated HDR, delivered into an AAV9, has been shown to mediate >1% HDR in the RPE of rd12 mice, which was sufficient to increase a- and b-wave responses (+21% and +40%, respectively) by ERG.^33^

CRISPR-Cas9-mediated HDR was also used in an X-linked RP murine model with a *Rpgr* mutation. Partial correction of *Rpgr* via CRISPR-Cas9-mediated HDR gene editing therapy improved photoreceptor survival.^34^ In an effort to increase HDR in post-mitotic cells, Cai *et al*. added a bacterial recombinase (RecA) to SpCas9 and used it to target *Pde6B* in postnatal rd1 mice using *in vivo* electroporation. They obtained a restoration of the expression of PDE6B in rod photoreceptors and an improved visual function in treated mice.^35^ These results demonstrate the potential of HDR by improved CRISPR-Cas9's variants but impose further challenges due to increased construct size by the addition of the RecA.

In the absence of homology-dependent recombination of template sequences, homology-independent targeted integration (HITI) can be used as an attractive alternative mechanism to knock in or promote correction of a sequence in IRDs. HITI is based on NHEJ-mediated targeted integration of a transgene.^36^ A donor DNA containing homologous arms matching the genomic locus of interest is inserted by the NHEJ pathway in between the flanking sites due to the DSB created by Cas9. When the donor DNA sequence is inserted in the correct orientation, it prevents further Cas9 cutting.

A HITI-AAV was designed to restore the photoreceptors' function in a rat model with a homozygous 1.9 kb deletion of intron 1 to exon 2 in the *Mertk* gene. The HITI-AAV contains a copy of *Mertk* exon 2 and a Cas9 with a gRNA targeting *Mertk* intron 1. Thereby, by leveraging HITI, the AAV will lead to an integration of a copy of *Mertk* exon 2 into intron 1. Subretinal injection leads to a 4.5% increase in *Mertk* mRNA expression levels, better preservation of the ONL thickness and significantly improved ERG b-wave responses.^36^ As HDR repair pathway is limited in post-mitotic cell, HITI offers an interesting alternative for insertion of a transgene in nondividing cells and might help advance basic and translational research in GT.

Lastly, microhomology-mediated end joining (MMEJ) can also be used as a repair pathway to mediate mutation replacement using CRISPR. MMEJ uses microhomologous sequences for error-prone end joining, resulting in deletions flanking the original break. It can be used as a “MMEJ-mediated gene knock-in strategy” to allow precise integration of a DNA donor in a desired genomic locus.^37^ For example, single-AAV2/8 delivery of CRISPR-SaCas9-MMEJ allows the corrected editing rate of 11% of *Gnat1* homozygous 59 bp deletion in rods in *Gnat1^IRD2/IRD2^/Pde6c^cpfl1/cpfl1^* mice with MMEJ-mediated mutation replacement. It leads to improvement in light sensitivity and partial ERG rescue.^38^

As previously mentioned, prolonged overexpression of CRISPR-Cas9 may lead to increased off-targets^27^ and immune responses.^28^ To this aim, variants of CRISPR-Cas9 with a restricted activity window have been developed, but only a few have been tested in the eye *in vivo*. One such example is a self-inactivating “kamikaze” CRISPR-Cas9 system. A second gRNA is added, in addition to the “target gRNA” and targets the SpCas9 itself. Such a system has been used in the IRDs after delivery by an AAV vector by intravitreal injection in transgenic mice and achieved high-efficiency genome editing and a decreased level of SpCas9 mRNA.^39^ Further studies should look at off-targets and immune response compared to regular Cas9.

Finally, catalytically inactive Cas9 (dead Cas9) can be fused with transcriptional repressors or activators to induce a transcriptional repression (CRISPR interference, or CRISPRi) or activation (CRISPRa).^40,41^ This system was used to repress *Nrl* in a mouse model of RP. *Nrl* knockdown mediates the reprogramming of rod cells into cone-like cells that are resistant to RP, with concomitant prevention of secondary cone loss.^42^ In conclusion, CRISPR-Cas9 improvements offer many possibilities for the treatment of IRDs but will need to be tested in larger animal models *in vivo* to determine their suitability for clinical use.

### BEs and PEs for the treatment of IRDs

Newer types of gene editing tools, called DNA BEs or PEs, allow the repair of point mutations without inducing a DSB.^43^ BEs are the fusion of CRISPR-Cas9 and a deaminase enzyme, which allow the direct conversion of a single nucleotide. Cytidine BEs convert C·G nucleotides into A·T nucleotides, and adenine BEs convert A·T nucleotides into C·G nucleotides.^44,45^ PEs consist of a Cas9 endonuclease fused to an engineered reverse transcriptase. They copy genetic information from a prime editing gRNA into a specific target genomic locus. Prime editors enable precise modification of all 12 possible classes of point mutations without requiring DSBs or donor DNA templates.^46^

PEs have less stringent protospacer adjacent motif requirements due to the varied length of the reverse transcription template compared to BEs and also no “bystander” edits.^47^ However, PEs have been less tested *in vivo* than BEs, and their specificity and potential for off-target modifications remain to be studied.

BEs have been efficiently delivered by subretinal injection via a dual AAV8 into photoreceptor cells, resulting in around 50% C·G-to-T·A editing efficiency among transduced rod photoreceptors.^48^ BEs were also used to correct a *Rpe65* mutation *in vivo* using lentiviral vectors. Correction of around 16% in the treated RPE tissue of rd12 mice restores the visual chromophore and rescues retinal and visual function (recovered average a- and b-wave amplitudes of 44% and 65% of the wt control responses, respectively).^49^

These tools are rapidly evolving. For example, an evolved BE minimizes even more the risk of off-targets. Bacterial selection was used to increase the stringency of the selection on modified TadA enzyme, contained in the BEs, to increase its activity.^50^

Also, thanks to fundamental research on CRISPR evolution, other RNA-guided nucleases called OMEGA have been discovered, suggesting that they are more common than previously suspected. Further research into their mechanism may lead to the development of even more powerful gene editing tools.^51^ In the future, these new editing tools will provide vast possibilities for the treatment of IRDs *in vivo*, even in post-mitotic cells.

However, one major limitation is their size, as they cannot be packaged into a single AAV. Indeed, as represented in Figure 1, in parallel with their optimization, new editors tend to gain size and exceed the carrying capacity of single AAVs, requiring the use of two dual AAVs. For example, a dual-AAV strategy was recently designed to deliver BE in neonatal mice.^52^ However, to limit a potential immune response, only half the dose of viral particles can be administered per vector, but both vectors need to transduce the same cell, limiting the potential efficacy. Moreover, with further increased sizes in BEs and PEs, even a dual-AAV strategy will be challenging. An important bottleneck is thus to find adequate delivery systems to transition their application into the clinic.

## Genes of Special Interest for GT Using CRISPR-Cas9

Gene addition is the most straightforward option for treating IRDs caused by a single recessive gene defect. In this type of treatment, addition of a normal copy of the gene into affected cells compensates for the loss of gene function due to a mutation. However, here we focus on the two main categories where gene addition using an AAV vector cannot be implemented due to either gain-of-function mutations leading to toxicity or the large size of the gene to be replaced. So, ar diseases due to mutations in big genes cannot be addressed by gene addition using a single AAV. Although dual or triple AAV systems are currently being evaluated to overcome this obstacle,^53^ gene editing tools offer a more promising alternative to treat these two types of diseases in the long run.

### Suppression and replacement for ad diseases

Ad diseases, which are caused by gain-of-function mutations leading to toxicity, cannot be addressed by gene addition therapy. GT for dominant-negative mutations is much more complex, since the causal mutation needs to be silenced.

In the eye, there is a particularly high number of ad diseases, accounting for 15–20% of all IRDs.^54^ adIRDs are characterized by an important genetic variability, with more than 1,000 variants reported^55^ and often with multiple mutations for each gene. In this context, it seems arduous to create a new targeting system using CRISPR-Cas9 for each mutation. One possibility to scale up the applicability of gene editing therapy is to perform biallelic knockout of the mutated gene. Healthy gene expression can complementarily be provided at the same time (suppression and replacement).

For example, more than 200 mutations have been reported in *RHO*, which is the most commonly mutated gene in adRP (accounting for around 30% in the United States and 20% in Europe).^56^ These mutations are referenced in the Human Gene Mutation Database (http://www.hgmd.cf.ac.uk/).^57^ Gain-of-function and/or dominant-negative mutations in *RHO* lead to a progressive loss of rods, resulting in a subsequent loss of cones and therefore progressive vision loss.

There is currently no treatment available for this disease, although Editas Medicine is currently developing a gene editing therapy using a dual AAV strategy: one AAV carries the SaCas9 to knock out the endogenous *RHO*, and the second AAV carries an exogenous *RHO* under a *RHO* promoter and the gRNA for the SaCas9. They obtained a 30% reduction in endogenous *hRHO* mRNA expression and up to 400 × replacement expression of exogenous *RHO* mRNA in mice with a knock-in of human *RHO*.^58^

However, this strategy of suppression and replacement will need to overcome multiple challenges in the coming years to be a viable therapy for patients.

As the ad diseases are due to a gain of function or a dominant-negative effect, the accumulation of the mutated protein is toxic to the cells. For mutations in *RHO* for example, multiple consequences of the defect can occur, and the toxic effect will generally be cumulative.^59^ In a suppress and replace strategy, the suppression by knockout will need to be sufficient to reverse or at least slow down the progression of the disease. However, thus far, CRISPR-Cas9 only reduces endogenous *hRHO* mRNA by 30% *in vivo* in a humanized mRho^hRHO/+^ mouse model.^58^

The same silence and replace strategy was developed before but using a shRNA for the silencing instead of the CRISPR-Cas9, and this obtained around the same efficacy, with 26% reduction in the hRHO transcript level (in a different model of humanized mice with mutation P347S).^60^ However, in a canine model of RHO-adRP, an 80% suppression of the endogenous canine *RHO* RNA was achieved,^61^ suggesting that, for now, shRNA might be more efficient than CRISPR-Cas9 in providing suppression. An important step will be to investigate if this decrease is sufficient to obtain a therapeutic effect.

Moreover, overexpression of *RHO* can be toxic.^62,63^ Therefore, the additional replacement of *RHO* will need to be tightly monitored, especially if the endogenous RHO is not completely knocked out. Furthermore, if the CRISPR-Cas9 cut is in a coding region, the exogenous *RHO* cDNA should be modified so that CRISPR-Cas9 does not cut the additional replacement of *RHO*. To avoid this problem, silent mutations can be introduced into the *RHO* cDNA.^61,63^ However, the silent mutations can decrease the rate of translation and the efficacy of the replacement.

### Mutation repair using BEs or PEs for arIRDs caused by mutations in large genes

As mentioned previously, gene supplementation is today the most commonly used GT for treating IRDs caused by a single recessive gene defect.

Currently, AAVs are the most efficient and safe gene delivery vectors. A dozen clinical trials are now exploring this strategy to improve clinical outcomes in patients affected with monogenic recessive diseases of the retina with known mutations.^64^ However, the packaging capacity of an AAV is limited to 4.7 kb, and several cis-regulatory elements need to be included in the transgene cassette besides the cDNA of the gene of interest. The gene of interest, with a promoter, is inserted between inverted terminal repeats (ITRs) alongside a polyadenylation signal. ITRs are each 145 bp (around 300 bp in total), short polyadenylation signals are around 200 bp, and the smaller promoters are around 500 bp. Even with the smallest elements, the size of the carried gene cannot exceed 3.9 kb.^65,66^

But the coding sequences of many genes involved in IRDs are larger and exceed this capacity of AAV (see Table 1). Lentiviruses have a bigger packaging capacity but have a very low photoreceptor transduction efficiency.^67^ Other GT solutions besides gene replacement using viral vectors should therefore be found to treat those arIRDs. For example, a correction of the mutation using editing technologies could be a solution.

**Table 1.** Disease-causing Genes Causing IRDs and of Interest for Gene Editing Therapy

Gene name	Transcript ID on Ensembl	Associated disease category on RetNet	CDS (bp)	Frequency
*CEP290*	ENST00000552810.6	Bardet–Biedl syndrome, ar	7,440	Rare^74^
*IFT172*	ENST00000260570.8	Bardet–Biedl syndrome, ar	5,250	Rare^75,76^
*ABCA4*	ENST00000370225.4	Cone or cone-rod dystrophy, ar	6,822	Frequent^77^
*CACNA1F*	ENST00000376265.2	Cone or cone-rod dystrophy, X-linked	5,934	Rare^78^
*GPR179*	ENST00000616987.5	CSNB, ar	7,104	Rare^79^
*TRPM1*	ENST00000397795.6	CSNB, ar	4,812	Frequent^79,80^
*CACNA1F*	ENST00000376265.2	CSNB, X-linked	5,934	Frequent^81,82^
*CEP290*	ENST00000552810.6	Leber congenital amaurosis, ar	7,440	Frequent^83^
*CRB1*	ENST00000367400.8	Leber congenital amaurosis, ar	4,221	Frequent^84^
*IFT140*	ENST00000426508.7	Leber congenital amaurosis, ar	4,389	Relatively rare^85^
*ABCA4*	ENST00000370225.4	Macular degeneration, ar	6,822	Frequent^86^
*ABCA4*	ENST00000370225.4	Retinitis pigmentosa, ar	6,822	Relatively rare^87^
*ARHGEF18*	ENST00000668164.2	Retinitis pigmentosa, ar	4,086	Rare^88^
*CRB1*	ENST00000367400.8	Retinitis pigmentosa, ar	4,221	Relatively rare^89^
*EYS*	ENST00000503581.6	Retinitis pigmentosa, ar	9,435	Relatively rare^90^
*GPR125*	ENST00000334304.10	Retinitis pigmentosa, ar	3,966	Rare^91^
*IFT172*	ENST00000260570.8	Retinitis pigmentosa, ar	5,250	Rare^91^
*KIAA1549*	ENST00000422774.2	Retinitis pigmentosa, ar	5,853	Rare^92^
*RP1*	ENST00000220676.2	Retinitis pigmentosa, ar	6,471	Relatively rare^87^
*RP1L1*	ENST00000382483.4	Retinitis pigmentosa, ar	7,203	Unknown
*USH2A*	ENST00000307340.8	Retinitis pigmentosa, ar	15,609	Frequent^93^
*ADGRV1*	ENST00000405460.9	Usher syndrome, ar	18,921	Relatively frequent^94^
*CDH23*	ENST00000224721.12	Usher syndrome, ar	10,065	Relatively frequent^94^
*CEP250*	ENST00000397527.6	Usher syndrome, ar	7,329	Relatively rare^95^
*MYO7A*	ENST00000409709.9	Usher syndrome, ar	6,648	Frequent^96^
*PCDH15*	ENST00000320301.11	Usher syndrome, ar	5,868	Rare^97^

Sort out from RetNet (https://sph.uth.edu/retnet/) according to these criteria: (1) genes >3.9 kb (do not fit into an adeno-associated virus for gene replacement); (2) nonsyndromic IRDs (due to its immune privilege and its relative isolation from other organs, the eye might be easier to target than other organs using intraocular injections); (3) autosomal recessive (ar) or X-linked IRDs (in autosomal dominant diseases caused by gain-of-function mutations, the toxic mutated protein needs to be silenced). Coding sequence (CDS) from Ensembl (https://www.ensembl.org/index.html).

IRD, inherited retinal dystrophy; CSNB, congenital stationary night blindness.

One possibility to induce a precise repair of the mutation is to use CRISPR-Cas9-mediated HITI, which generates a DNA knock-in, even in nondividing cells. A transgene can be integrated at a specific site to repair a mutation for a potential GT. However, CRISPR-Cas9-mediated HITI efficacy *in vivo* in the retina is currently limited. In a rat model, a CRISPR-Cas9-mediated HITI induces a 4.5% increase in the mRNA expression levels of the gene of interest and with only a partial restoration of vision.^36^ Moreover, the DSB generated by CRISPR-Cas9 will also activate other repair pathways, leading to indels, and also carries the risk of off-targets.

Newer editing tools, namely BEs and PEs, enable precise and targeted nucleotide substitutions without inducing a DSB.^44–46^ BEs can introduce all four transition mutations, and PEs can perform all 12 possible transition and transversion mutations as well as small indel mutations. They offer real hope as therapeutic tools to correct disease-causing mutations in IRDs.

Certain candidate genes for GT using BEs or PEs might be more suitable to start with. IRDs, being a heterogenous group of diseases, and some other diseases might be simpler to treat for initial proof-of-concept studies.

First, IRDs can affect only the eye or can be syndromic. Due to its immune privilege and its relative isolation from other organs, the eye might be easier to target than other organs using intraocular injections. Therefore, we here focus only on nonsyndromic retinal dystrophies (Table 1).

Furthermore, to select good candidates for GT using BEs or PEs, it is important to consider the age of onset and the rate of degeneration of the disease. Gene editing should be implemented as soon as possible after the genetic diagnosis of the disease. It will be easier, at least at the beginning, to target diseases that have a late age of onset and are nonprogressive or with a relatively slow rate of degeneration. However, if the gene editing is very efficient, it might be possible to overcome these challenges. For example, EDIT-101 is now being tested in a Phase I/II clinical trial (NCT03872479), even though it targets the *CEP290* gene leading to LCA10, where the age of onset is very early.^25^

Stargardt disease (STGD) is considered to be a less severe disease than cone-rod dystrophy or RP because of the retina-wide involvement implicit in the latter diagnoses. Mutations in *ABCA4* are a major cause of STGD, and even though they cover a wide spectrum of severity, most individuals with *ABCA4* disease have an intermediate phenotype between the extremes.^68^ The missense mutation c.5882G>A, p.(G1961E) is the most common mutation of *ABCA4* (18.5% in Europe and the United States).^69,70^ BE or PEs could be used to reverse this mutation to stop or slow down the degeneration of patients' retinas. But BEs and PEs are recently developed tools that have not been tested widely *in vivo* in the eye, and one of the main challenges will be to deliver them because of their size.

In the meantime, other GTs are being developed for *ABCA4* mutations in patients with Stargardt's macular degeneration. A lentivirus gene addition is currently in Phase I/II of a clinical trial (NCT01736592), and another clinical trial using a dual AAV may follow soon.^71^ Gene editing therapies will have to be more effective and less toxic than these other GT solutions to find their place in the GT landscape.

In addition, to select good candidates for GT using BEs or PEs, the cell types affected by the disease should be carefully considered. Most forms of IRDs mainly affect photoreceptors, but some forms can also affect the RPE or other retinal cell types. It would be easier to target a gene expressed in the RPE than in the retina. Indeed, when injected subretinally, RPE cells absorb more vectors than retinal cells because of the properties of the cell type: it is a monolayer of cells, there is no barrier as the outer limiting membrane, and the cells are less compacted than retinal cells. AAV expression is therefore more pronounced in the RPE, and the efficacy of CRISPR is generally higher. Nevertheless, IRDs mainly affect photoreceptors,^72^ and only a few genes that are expressed in the RPE are known to cause IRDs.

Among them, *RPE65*, *LRAT*, *RLBP1*, and *RDH5* are <3.9 kb. Gene addition using AAV vectors, that is currently at a more advanced stage of development for the clinic than gene editing, would therefore be more suitable for arIRDs with mutations in one of these genes. Indeed, RP and LCA associated with mutations in *RPE65* benefit from the Luxturna gene augmentation therapy.

An important criterion to select a candidate gene for gene editing therapy will be not only the possibility of another GT, but also the frequency of the gene mutation and the heterogeneity of the mutations within the same gene.

Many IRDs could benefit from BEs or PEs in the future. For example, *USH2A* or *ADGRV1* coding sequences are 15,609 and 19,557 bp, respectively, and will never fit into even a dual AAV. However, these tools are recent, and their efficacy and potential toxicity will need to be more evaluated *in vivo* in the eye before going to the clinic. In particular, although BEs and PEs offer a reduced off-target risk, efforts are still needed to narrow the editing window to reduce by products.

## Discussion/Conclusion

Precise editing tools, such as CRISPR-Cas9, are emerging as a potential solution for GT and are opening up incredible possibilities for future treatments of IRDs. CRISPR-Cas9 has been tested in multiple animal models for multiple IRDs and will become an important GT solution in the coming years.

First, CRISPR-Cas9 is exploitable to remove an aberrant splice donor and to restore the activity of a given protein. The main advanced example of it is EDIT-101, which targets an aberrant splice donor created by the IVS26 mutation in *CEP290* leading to LCA10 and is now being tested in a Phase I/II clinical trial (NCT03872479). Dosing of the adult cohort has been completed, and the trial is now enrolling its first pediatric patients (from ir.editasmedicine.com, Results and Business Updates, August 4, 2021).

Also, ad diseases, which cannot benefit from a simple gene addition due to the toxic effect of the mutated protein, could benefit from CRISPR-Cas9 by knocking down the endogenous mutated gene and replacing it with an exogenous wt (silence and replace).

Many other IRDs might benefit from gene editing therapy, as the CRISPR-Cas9 toolbox is expanding rapidly, in particular with BEs and PEs. They could be an alternative solution to target mutations in genes that are >3.9 kb and do not fit into a single AAV for a gene addition strategy. However, for now, other options, currently more advanced, are available to overcome this issue of limited size packaging of the AAV. For example, dual AAV systems, lentivirus, and minigenes are currently being developed. Smaller Cas are also being optimized.^73^

Gene editing therapy will have to face many challenges before improving patients' lives. Important efforts should be made to increase the efficacy of the CRISPR-Cas9 system targeting the retina and to reduce its risks, with specific attention to off-targets and byproducts. Optimization of the editor's vectorization will also be important for future clinical applications. AAV have shown some limits, with potential immunogenicity and increased risk of off-targets due to the long-term expression of Cas9 derivatives. Consequently, nonviral vectors might emerge as an interesting solution if important upgrades are made to increase their low efficacy toward neural tissue.

Gene editing technologies will have to prove better efficacy and reduce risks to become a realistic and suitable GT solution in the coming years. With a parallel development of the gene editing and delivery technologies, these improvements look achievable in the foreseeable future.

## References

[1] Sahel J, Marazova K, Audo I. Clinical characteristics and current therapies for inherited retinal degenerations. Cold Spring Harb Perspect Med 2014;5:a017111. DOI: 10.1101/CSHPERSPECT.A017111. 25324231 PMC4315917

[2] Bainbridge JWB, Smith AJ, Barker SS, et al. Effect of gene therapy on visual function in Leber's congenital amaurosis. New Engl J Med 2008;358:2231–2239. DOI: 10.1056/NEJMOA0802268/SUPPL_FILE/NEJM_BAINBRIDGE_2231SA1.PDF. 18441371

[3] Maguire AM, Simonelli F, Pierce EA, et al. Safety and efficacy of gene transfer for Leber's congenital amaurosis. New Engl J Med 2008;358:2240–2248. DOI: 10.1056/NEJMOA0802315/SUPPL_FILE/NEJM_MAGUIRE_2240SA1.PDF. 18441370 PMC2829748

[4] Gaj T, Gersbach CA, Barbas CF III. ZFN, TALEN and CRISPR/Cas-based methods for genome engineering. Trends Biotechnol 2013;31:397. DOI: 10.1016/J.TIBTECH.2013.04.004. 23664777 PMC3694601

[5] Chandrasegaran S, Carroll D. Origins of programmable nucleases for genome engineering. J Mol Biol 2016;428:963–989. DOI: 10.1016/J.JMB.2015.10.014. 26506267 PMC4798875

[6] Hirakawa MP, Krishnakumar R, Timlin JA, et al. Gene editing and CRISPR in the clinic: current and future perspectives. Biosci Rep 2020;40:BSR20200127. DOI: 10.1042/BSR20200127. 32207531 PMC7146048

[7] Low BE, Krebs MP, Joung JK, et al. Correction of the Crb1rd8 allele and retinal phenotype in C57BL/6N mice via TALEN-mediated homology-directed repair. Invest Ophthalmol Vis Sci 2013;55:387–395. DOI: 10.1167/iovs.13-13278. PMC3921157 24346171

[8] Joung JK, Sander JD. TALENs: a widely applicable technology for targeted genome editing. Nat Rev Mol Cell Biol 2012;14:49–55. DOI: 10.1038/NRM3486. 23169466 PMC3547402

[9] Cong L, Ran FA, Cox D, et al. Multiplex genome engineering using CRISPR/Cas systems. Science 2013;339:819–823. DOI: 10.1126/science.1231143. 23287718 PMC3795411

[10] Jinek M, Chylinski K, Fonfara I, et al. A programmable dual-RNA-guided DNA endonuclease in adaptive bacterial immunity. Science 2012;337:816–821. DOI: 10.1126/science.1225829. 22745249 PMC6286148

[11] Pickar-Oliver A, Gersbach CA. The next generation of CRISPR-Cas technologies and applications. Nat Rev Mol Cell Biol 2019;20:490–507. DOI: 10.1038/S41580-019-0131-5. 31147612 PMC7079207

[12] Doench J, Fusi N, Sullender M, et al. Optimized sgRNA design to maximize activity and minimize off-target effects of CRISPR-Cas9. Nat Biotechnol 2016;34:184–191. DOI: 10.1038/NBT.3437. 26780180 PMC4744125

[13] Li F, Wing K, Wang JH, et al. Comparison of CRISPR/Cas Endonucleases for *in vivo* retinal gene editing. Front Cell Neurosci 2020;14:570917. DOI: 10.3389/fncel.2020.570917. 33132845 PMC7511709

[14] Bakondi B, Lv W, Lu B, et al. *In vivo* CRISPR/Cas9 gene editing corrects retinal dystrophy in the S334ter-3 rat model of autosomal dominant retinitis pigmentosa. Mol Ther 2016;24:556–563. DOI: 10.1038/mt.2015.220. 26666451 PMC4786918

[15] Latella MC, Di Salvo MT, Cocchiarella F, et al. *In vivo* editing of the human mutant rhodopsin gene by electroporation of plasmid-based CRISPR/Cas9 in the mouse retina. Mol Ther Nucleic Acids 2016;5:e389. DOI: 10.1038/mtna.2016.92. 27874856 PMC5155324

[16] Li P, Kleinstiver BP, Leon MY, et al. Allele-specific CRISPR-Cas9 genome editing of the single-base P23H mutation for rhodopsin-associated dominant retinitis pigmentosa. CRISPR J 2018;1:55–64. DOI: 10.1089/crispr.2017.0009. 31021187 PMC6319323

[17] Giannelli SG, Luoni M, Castoldi V, et al. Cas9/sgRNA selective targeting of the P23H rhodopsin mutant allele for treating retinitis pigmentosa by intravitreal AAV9.PHP.B-based delivery. Hum Mol Genet 2018;27:761–779. DOI: 10.1093/hmg/ddx438. 29281027

[18] Patrizi C, Llado M, Benati D, et al. Allele-specific editing ameliorates dominant retinitis pigmentosa in a transgenic mouse model. Am J Hum Genet 2021;108:295–308. DOI: 10.1016/j.ajhg.2021.01.006. 33508235 PMC7896132

[19] Tsai Y-T, Wu W-H, Lee T-T, et al. Clustered regularly interspaced short palindromic repeats–based genome surgery for the treatment of autosomal dominant retinitis pigmentosa. Ophthalmology 2018;125:1421–1430. DOI: 10.1016/j.ophtha.2018.04.001. 29759820 PMC6109419

[20] Wu W-H, Tsai Y-T, Huang I-W, et al. CRISPR genome surgery in a novel humanized model for autosomal dominant retinitis pigmentosa. Mol Ther 2022 Feb 10 [Epub ahead of print]; DOI: 10.1016/J.YMTHE.2022.02.010. 10.1016/j.ymthe.2022.02.010 PMC9077379 35150888

[21] Karapurkar JK, Antao AM, Kim KS, et al. CRISPR-Cas9 based genome editing for defective gene correction in humans and other mammals. Prog Mol Biol Transl Sci 2021;181:185–229. DOI: 10.1016/bs.pmbts.2021.01.018. 34127194

[22] Yu W, Mookherjee S, Chaitankar V, et al. Nrl knockdown by AAV-delivered CRISPR/Cas9 prevents retinal degeneration in mice. Nat Commun 2017;8:14716. DOI: 10.1038/ncomms14716. 28291770 PMC5355895

[23] Zhu J, Ming C, Fu X, et al. Gene and mutation independent therapy via CRISPR-Cas9 mediated cellular reprogramming in rod photoreceptors. Cell Res 2017;27:830–833. DOI: 10.1038/cr.2017.57. 28429769 PMC5518875

[24] McCullough KT, Boye SL, Fajardo D, et al. Somatic gene editing of GUCY2D by AAV-CRISPR/Cas9 alters retinal structure and function in mouse and macaque. Hum Gene Ther 2019;30:571–589. DOI: 10.1089/hum.2018.193. 30358434 PMC6534089

[25] Maeder ML, Stefanidakis M, Wilson CJ, et al. Development of a gene-editing approach to restore vision loss in Leber congenital amaurosis type 10. Nat Med 2019;25:229–233. DOI: 10.1038/s41591-018-0327-9. 30664785

[26] Hanlon KS, Kleinstiver BP, Garcia SP, et al. High levels of AAV vector integration into CRISPR-induced DNA breaks. Nat Commun 2019;10:4439. DOI: 10.1038/s41467-019-12449-2. 31570731 PMC6769011

[27] Sander JD, Joung JK. CRISPR-Cas systems for editing, regulating and targeting genomes. Nat Biotechnol 2014;32:347–350. DOI: 10.1038/nbt.2842. 24584096 PMC4022601

[28] Charlesworth CT, Deshpande PS, Dever DP, et al. Identification of preexisting adaptive immunity to Cas9 proteins in humans. Nat Med 2019;25:249–254. DOI: 10.1038/s41591-018-0326-x. 30692695 PMC7199589

[29] van Haasteren J, Li J, Scheideler OJ, et al. The delivery challenge: fulfilling the promise of therapeutic genome editing. Nat Biotechnol 2020;38:845–855. DOI: 10.1038/S41587-020-0565-5. 32601435

[30] Kim K, Park SW, Kim JH, et al. Genome surgery using Cas9 ribonucleoproteins for the treatment of age-related macular degeneration. Genome Res 2017;27:419–426. DOI: 10.1101/gr.219089.116. 28209587 PMC5340969

[31] Holmgaard AB, Askou AL, Jensen EG, et al. Targeted knockout of the *Vegfa* gene in the retina by subretinal injection of RNP complexes containing Cas9 protein and modified sgRNAs. Mol Ther 2021;29:191–207. DOI: 10.1016/j.ymthe.2020.09.032. 33022212 PMC7791085

[32] Chen G, Abdeen AA, Wang Y, et al. A biodegradable nanocapsule delivers a Cas9 ribonucleoprotein complex for *in vivo* genome editing. Nat Nanotechnol 2019;14:974–980. DOI: 10.1038/s41565-019-0539-2. 31501532 PMC6778035

[33] Jo DH, Song DW, Cho CS, et al. CRISPR-Cas9–mediated therapeutic editing of Rpe65 ameliorates the disease phenotypes in a mouse model of Leber congenital amaurosis. Sci Adv 2019;5:eaax1210. DOI: 10.1126/sciadv.aax1210. 31692906 PMC6821465

[34] Hu S, Du J, Chen N, et al. *In vivo* CRISPR/Cas9-mediated genome editing mitigates photoreceptor degeneration in a mouse model of X-linked retinitis pigmentosa. Invest Ophthalmol Vis Sci 2020;61:31. DOI: 10.1167/iovs.61.4.31. PMC7401909 32330228

[35] Cai Y, Cheng T, Yao Y, et al. *In vivo* genome editing rescues photoreceptor degeneration via a Cas9/RecA-mediated homology-directed repair pathway. Sci Adv 2019;5:eaav3335. 10.1126/sciadv.aav3335. 31001583 PMC6469935

[36] Suzuki K, Tsunekawa Y, Hernandez-Benitez R, et al. *In vivo* genome editing via CRISPR/Cas9 mediated homology-independent targeted integration. Nature 2016;540:144–149. DOI: 10.1038/nature20565. 27851729 PMC5331785

[37] Nakade S, Tsubota T, Sakane Y, et al. Microhomology-mediated end-joining-dependent integration of donor DNA in cells and animals using TALENs and CRISPR/Cas9. Nat Commun 2014;5:5560. DOI: 10.1038/ncomms6560. 25410609 PMC4263139

[38] Nishiguchi KM, Fujita K, Miya F, et al. Single AAV-mediated mutation replacement genome editing in limited number of photoreceptors restores vision in mice. Nat Commun 2020;11:482. DOI: 10.1038/s41467-019-14181-3. 31980606 PMC6981188

[39] Li F, Hung SSC, Mohd Khalid MKN, et al. Utility of self-destructing CRISPR/Cas constructs for targeted gene editing in the retina. Hum Gene Ther 2019;30:1349–1360. DOI: 10.1089/hum.2019.021. 31373227

[40] Qi LS, Larson MH, Gilbert LA, et al. Repurposing CRISPR as an RNA-guided platform for sequence-specific control of gene expression. Cell 2013;152:1173–1183. DOI: 10.1016/j.cell.2013.02.022. 23452860 PMC3664290

[41] Dominguez AA, Lim WA, Qi LS. Beyond editing: repurposing CRISPR-Cas9 for precision genome regulation and interrogation. Nat Rev Mol Cell Biol 2016;17:5–15. DOI: 10.1038/nrm.2015.2. 26670017 PMC4922510

[42] Moreno AM, Fu X, Zhu J, et al. *In situ* gene therapy via AAV-CRISPR-Cas9-mediated targeted gene regulation. Mol Ther 2018;26:1818–1827. DOI: 10.1016/j.ymthe.2018.04.017. 29754775 PMC6035733

[43] Anzalone AV, Koblan LW, Liu DR. Genome editing with CRISPR-Cas nucleases, base editors, transposases and prime editors. Nat Biotechnol 2020;38:824–844. DOI: 10.1038/S41587-020-0561-9. 32572269

[44] Komor AC, Kim YB, Packer MS, et al. Programmable editing of a target base in genomic DNA without double-stranded DNA cleavage. Nature 2016;533:420–424. DOI: 10.1038/nature17946. 27096365 PMC4873371

[45] Gaudelli NM, Komor AC, Rees HA, et al. Programmable base editing of T to G C in genomic DNA without DNA cleavage. Nature 2017;551:464–471. DOI: 10.1038/nature24644. 29160308 PMC5726555

[46] Anzalone AA V, Randolph PB, Davis JR, et al. Search-and-replace genome editing without double-strand breaks or donor DNA. Nature 2019;576:149–157. DOI: 10.1038/s41586-019-1711-4. 31634902 PMC6907074

[47] Kantor A, McClements ME, Maclaren RE. CRISPR-Cas9 DNA base-editing and prime-editing. Int J Mol Sci 2020;21:1–22. DOI: 10.3390/IJMS21176240. PMC7503568 32872311

[48] Levy JM, Yeh WH, Pendse N, et al. Cytosine and adenine base editing of the brain, liver, retina, heart and skeletal muscle of mice via adeno-associated viruses. Nat Biomed Eng 2020;4:97–110. DOI: 10.1038/s41551-019-0501-5. 31937940 PMC6980783

[49] Suh S, Choi EH, Leinonen H, et al. Restoration of visual function in adult mice with an inherited retinal disease via adenine base editing. Nat Biomed Eng 2021;5:169–178. DOI: 10.1038/s41551-020-00632-6. 33077938 PMC7885272

[50] Gaudelli NM, Lam DK, Rees HA, et al. Directed evolution of adenine base editors with increased activity and therapeutic application. Nat Biotechnol 2020;38:892–900. DOI: 10.1038/S41587-020-0491-6. 32284586

[51] Altae-Tran H, Kannan S, Demircioglu FE, et al. The widespread IS200/605 transposon family encodes diverse programmable RNA-guided endonucleases. Science 2021;374:57–65. DOI: 10.1126/science.abj6856. 34591643 PMC8929163

[52] Zhou L, Su J, Long J, et al. A universal strategy for AAV delivery of base editors to correct genetic point mutations in neonatal PKU mice. Mol Ther Methods Clin Dev 2022;24:230–240. DOI: 10.1016/j.omtm.2022.01.001. 35141352 PMC8803597

[53] Trapani I, Tornabene P, Auricchio A. Large gene delivery to the retina with AAV vectors: are we there yet? Gene Ther 2020;28:220–222. DOI: 10.1038/S41434-020-0174-4. 32661283

[54] Perea-Romero I, Gordo G, Iancu IF, et al. Genetic landscape of 6089 inherited retinal dystrophies affected cases in Spain and their therapeutic and extended epidemiological implications. Sci Rep 2021;11:1526. DOI: 10.1038/s41598-021-81093-y. 33452396 PMC7810997

[55] Hanany M, Sharon D. Allele frequency analysis of variants reported to cause autosomal dominant inherited retinal diseases question the involvement of 19% of genes and 10% of reported pathogenic variants. J Med Genet 2019;56:536–542. DOI: 10.1136/jmedgenet-2018-105971. 30910914

[56] Diakatou M, Manes G, Bocquet B, Meunier I, Kalatzis V. Genome editing as a treatment for the most prevalent causative genes of autosomal dominant retinitis pigmentosa. Int J Mol Sci 2019;20:2542. DOI: 10.3390/ijms20102542. PMC6567127 31126147

[57] Stenson PD, Mort M, Ball EV, et al. The Human Gene Mutation Database: building a comprehensive mutation repository for clinical and molecular genetics, diagnostic testing and personalized genomic medicine. Hum Genet 2014;133:1–9. DOI: 10.1007/S00439-013-1358-4. 24077912 PMC3898141

[58] Allocca M, Liu C, Nayak R, et al. Advances toward a dual AAV CRISPR-Cas9-based “knockout and replace” strategy to treat rhodopsin-associated autosomal dominant retinitis pigmentosa. Available online at: https://www.editasmedicine.com/wp-content/uploads/2021/04/ARVO-2021-Poster_Rho-adRP_FINAL.pdf (last accessed April 11, 2022).

[59] Athanasiou D, Aguila M, Bellingham J, et al. The molecular and cellular basis of rhodopsin retinitis pigmentosa reveals potential strategies for therapy. Prog Retin Eye Res 2018;62:1–23. DOI: 10.1016/j.preteyeres.2017.10.002. 29042326 PMC5779616

[60] Mussolino C, Sanges D, Marrocco E, et al. Zinc-finger-based transcriptional repression of rhodopsin in a model of dominant retinitis pigmentosa. EMBO Mol Med 2011;3:118–128. DOI: 10.1002/emmm.201000119. 21268285 PMC3085076

[61] Cideciyan A V., Sudharsan R, Dufour VL, et al. Mutation-independent rhodopsin gene therapy by knockdown and replacement with a single AAV vector. Proc Natl Acad Sci U S A 2018;115:E8547–E8556. DOI: 10.1073/pnas.1805055115. 30127005 PMC6130384

[62] Tan E, Wang Q, Quiambao AB, et al. The relationship between opsin overexpression and photoreceptor degeneration. Invest Ophthalmol Vis Sci 2001;42:589–600. 11222515

[63] Mao H, James T, Schwein A, et al. AAV delivery of wild-type rhodopsin preserves retinal function in a mouse model of autosomal dominant retinitis pigmentosa. Hum Gene Ther 2011;22:567–575. DOI: 10.1089/hum.2010.140. 21126223 PMC3131806

[64] Bennett J. Taking stock of retinal gene therapy: looking back and moving forward. Mol Ther 2017;25:1076–1094. DOI: 10.1016/j.ymthe.2017.03.008. 28391961 PMC5417792

[65] Trapani I. Adeno-associated viral vectors as a tool for large gene delivery to the retina. Genes 2019;10. DOI: 10.3390/GENES10040287. PMC6523333 30970639

[66] Hirsch M, Wolfe SA, Samulski RJ. Delivering transgenic DNA exceeding the carrying capacity of AAV vectors. Methods Mol Biol 2016;1382:21–39. DOI: 10.1007/978-1-4939-3271-9_2. 26611576 PMC4971574

[67] Auricchio A, Trapani I, Allikmets R. Gene therapy of ABCA4-associated diseases. Cold Spring Harb Perspect Med 2015;5:a017301. DOI: 10.1101/CSHPERSPECT.A017301. 25573774 PMC4448589

[68] Cideciyan A V., Swider M, Aleman TS, et al. ABCA4 disease progression and a proposed strategy for gene therapy. Hum Mol Genet 2009;18:931. DOI: 10.1093/HMG/DDN421. 19074458 PMC2640207

[69] Rozet JM, Gerber S, Souied E, et al. The *ABCR* gene: a major disease gene in macular and peripheral retinal degenerations with onset from early childhood to the elderly. Mol Genet Metab 1999;68:310–315. DOI: 10.1006/MGME.1999.2925. 10527682

[70] Burke TR, Fishman GA, Zernant J, et al. Retinal phenotypes in patients homozygous for the G1961E mutation in the *ABCA4* gene. Invest Ophthalmol Vis Sci 2012;53:4458–4467. DOI: 10.1167/IOVS.11-9166. 22661473 PMC3394687

[71] Trapani I. Dual AAV vectors for Stargardt disease. Methods Mol Biol 2018;1715:153–175. DOI: 10.1007/978-1-4939-7522-8_11. 29188512

[72] Scholl HPN, Strauss RW, Singh MS, et al. Emerging therapies for inherited retinal degeneration. Sci Transl Med 2016;8:368rv6. DOI: 10.1126/SCITRANSLMED.AAF2838. 27928030

[73] Xu X, Chemparathy A, Zeng L, et al. Engineered miniature CRISPR-Cas system for mammalian genome regulation and editing. Mol Cell 2021;81:4333–4345.e4. DOI: 10.1016/J.MOLCEL.2021.08.008. 34480847

[74] Leitch CC, Zaghloul NA, Davis EE, et al. Hypomorphic mutations in syndromic encephalocele genes are associated with Bardet–Biedl syndrome. Nat Genet 2008;40:443–448. DOI: 10.1038/NG.97. 18327255

[75] Aldahmesh MA, Li Y, Alhashem A, et al. IFT27, encoding a small GTPase component of IFT particles, is mutated in a consanguineous family with Bardet–Biedl syndrome. Hum Mol Genet 2014;23:3307–3315. DOI: 10.1093/HMG/DDU044. 24488770 PMC4047285

[76] Bujakowska KM, Zhang Q, Siemiatkowska AM, et al. Mutations in IFT172 cause isolated retinal degeneration and Bardet–Biedl syndrome. Hum Mol Genet 2015;24:230–242. DOI: 10.1093/HMG/DDU441. 25168386 PMC4326328

[77] Hamel CP. Cone rod dystrophies. Orphanet J Rare Dis 2007;2:1–7. DOI: 10.1186/1750-1172-2-7. 17270046 PMC1808442

[78] Hameed A, Abid A, Aziz A, et al. Evidence of RPGRIP1 gene mutations associated with recessive cone-rod dystrophy. J Med Genet 2003;40:616–619. DOI: 10.1136/JMG.40.8.616. 12920076 PMC1735563

[79] Audo I, Bujakowska K, Orhan E, et al. Whole-exome sequencing identifies mutations in GPR179 leading to autosomal-recessive complete congenital stationary night blindness. Am J Hum Genet 2012;90:321–330. DOI: 10.1016/J.AJHG.2011.12.007. 22325361 PMC3276675

[80] AlTalbishi A, Zelinger L, Zeitz C, et al. *TRPM1* mutations are the most common cause of autosomal recessive congenital stationary night blindness (CSNB) in the Palestinian and Israeli populations. Sci Rep 2019;9:1–6. DOI: 10.1038/s41598-019-46811-7. 31427709 PMC6700182

[81] Strom TM, Nyakatura G, Apfelstedt-Sylla E, et al. An L-type calcium-channel gene mutated in incomplete X-linked congenital stationary night blindness. Nat Genet 1998;19:260–263. DOI: 10.1038/940. 9662399

[82] Bech-Hansen NT, Naylor MJ, Maybaum TA, et al. Loss-of-function mutations in a calcium-channel α1-subunit gene in Xp11.23 cause incomplete X-linked congenital stationary night blindness. Nat Genet 1998;19:264–267. DOI: 10.1038/947. 9662400

[83] Den Hollander AI, Koenekoop RK, Yzer S, et al. Mutations in the *CEP290* (*NPHP6*) gene are a frequent cause of Leber congenital amaurosis. Am J Hum Genet 2006;79:556–561. DOI: 10.1086/507318. 16909394 PMC1559533

[84] Saberi M, Golchehre Z, Karamzade A, et al. CRB1-related Leber congenital amaurosis: reporting novel pathogenic variants and a brief review on mutations spectrum. Iranian Biomed J 2019;23:362. DOI: 10.29252/.23.5.362. PMC6661128 31103025

[85] Xu M, Yang L, Wang F, et al. Mutations in human IFT140 cause non-syndromic retinal degeneration. Hum Genet 2015;134:1069–1078. DOI: 10.1007/S00439-015-1586-X. 26216056 PMC4565766

[86] Allikmets R, Shroyer NF, Singh N, et al. Mutation of the Stargardt Disease Gene (ABCR) in age-related macular degeneration. Science 1997;277:1805–1807. DOI: 10.1126/SCIENCE.277.5333.1805. 9295268

[87] Ferrari S, Di Iorio E, Barbaro V, et al. Retinitis pigmentosa: genes and disease mechanisms. Curr Genom 2011;12:238. DOI: 10.2174/138920211795860107. PMC3131731 22131869

[88] Arno G, Carss KJ, Hull S, et al. Biallelic mutation of *ARHGEF18*, involved in the determination of epithelial apicobasal polarity, causes adult-onset retinal degeneration. Am J Hum Genet 2017;100:334–342. DOI: 10.1016/J.AJHG.2016.12.014. 28132693 PMC5294887

[89] Corton M, Tatu SD, Avila-Fernandez A, et al. High frequency of *CRB1* mutations as cause of early-onset retinal dystrophies in the Spanish population. Orphanet J Rare Dis 2013;8:1–12. DOI: 10.1186/1750-1172-8-20. 23379534 PMC3637806

[90] Littink KW, van den Born LI, Koenekoop RK, et al. Mutations in the *EYS* gene account for approximately 5% of autosomal recessive retinitis pigmentosa and cause a fairly homogeneous phenotype. Ophthalmology 2010;117:2026–2033.e7. DOI: 10.1016/J.OPHTHA.2010.01.040. 20537394

[91] Abu-Safieh L, Alrashed M, Anazi S, et al. Autozygome-guided exome sequencing in retinal dystrophy patients reveals pathogenetic mutations and novel candidate disease genes. Genome Res 2013;23:236–247. DOI: 10.1101/GR.144105.112. 23105016 PMC3561865

[92] Huang L, Zhang Q, Huang X, et al. Mutation screening in genes known to be responsible for retinitis pigmentosa in 98 small Han Chinese families. Sci Rep 2017;7:1948. DOI: 10.1038/S41598-017-00963-6. 28512305 PMC5434011

[93] Toualbi L, Toms M, Moosajee M. USH2A-retinopathy: from genetics to therapeutics. Exp Eye Res 2020;201:108330. DOI: 10.1016/J.EXER.2020.108330. 33121974 PMC8417766

[94] Jouret G, Poirsier C, Spodenkiewicz M, et al. Genetics of Usher syndrome: new insights from a meta-analysis. Otol Neurotol 2019;40:121–129. DOI: 10.1097/MAO.0000000000002054. 30531642

[95] Fuster-García C, García-García G, Jaijo T, et al. High-throughput sequencing for the molecular diagnosis of Usher syndrome reveals 42 novel mutations and consolidates CEP250 as Usher-like disease causative. Sci Rep 2018;8:17113. DOI: 10.1038/S41598-018-35085-0. 30459346 PMC6244211

[96] Cheng L, Yu H, Jiang Y, et al. Identification of a novel MYO7A mutation in Usher syndrome type 1. Oncotarget 2018;9:2295. DOI: 10.18632/ONCOTARGET.23408. 29416772 PMC5788640

[97] Ahmed ZM, Riazuddin S, Bernstein SL, et al. Mutations of the protocadherin gene PCDH15 cause Usher syndrome type 1F. Am J Hum Genet 2001;69:25. DOI: 10.1086/321277. 11398101 PMC1226045

[98] Richter MF, Zhao KT, Eton E, et al. Phage-assisted evolution of an adenine base editor with improved Cas domain compatibility and activity. Nat Biotechnol 2020;38:883–891. DOI: 10.1038/s41587-020-0453-z. 32433547 PMC7357821

[99] Huang TP, Zhao KT, Miller SM, et al. Circularly permuted and PAM-modified Cas9 variants broaden the targeting scope of base editors. Nat Biotechnol 2019;37:626–631. DOI: 10.1038/S41587-019-0134-Y. 31110355 PMC6551276

